# SGLT-2 Inhibitor-Induced Euglycemic Diabetic Ketoacidosis Masked by Concurrent Pneumoperitoneum Following Spinal Surgery Under General Anesthesia: A Case Report

**DOI:** 10.3390/jcm15166145

**Published:** 2026-08-07

**Authors:** Minju Kim, Jiyoon Bhan, Do Gyeong Lee, Hyun Sik Chung

**Affiliations:** Department of Anesthesiology and Pain Medicine, Seoul St. Mary’s Hospital, College of Medicine, The Catholic University of Korea, Seoul 06591, Republic of Korea; minju1025@catholic.ac.kr (M.K.); jiyun970711@naver.com (J.B.); drdgcatholic@gmail.com (D.G.L.)

**Keywords:** SGLT-2 inhibitor, euglycemic diabetic ketoacidosis, perioperative, direct lateral interbody fusion, empagliflozin

## Abstract

**Background**: Sodium–glucose co-transporter-2 (SGLT-2) inhibitors are widely prescribed for type 2 diabetes mellitus (T2DM) because of their cardiovascular and renoprotective benefits. However, their use is associated with euglycemic diabetic ketoacidosis (EDKA), a rare but potentially life-threatening complication characterized by severe ketoacidosis despite relatively normal blood glucose levels. Failure to discontinue SGLT-2 inhibitors before surgery, as recommended in current guidelines, together with perioperative fasting and surgical stress, increases the risk of EDKA. Diagnostic complexity is compounded when concurrent postoperative surgical complications provide an alternative explanation for persistent metabolic acidosis. **Methods**: A 71-year-old man with T2DM receiving uninterrupted empagliflozin underwent direct lateral interbody fusion under general anesthesia. On postoperative day 2, he developed severe high anion-gap metabolic acidosis (pH 7.204, HCO_3_^−^ 10.1 mEq/L) with near-normal blood glucose levels (178 mg/dL). Pneumoperitoneum identified on imaging was attributed to Hemovac drain-related peritoneal injury, and emergent laparoscopic exploration was performed under a working diagnosis of surgical sepsis. Although surgical source control was successfully achieved, severe metabolic acidosis persisted postoperatively (pH 7.275). Euglycemic diabetic ketoacidosis is an uncommon diabetic complication associated with several perioperative risk factors, including prolonged fasting and surgical stress. Subsequent serum ketone analysis demonstrated markedly elevated beta-hydroxybutyrate levels (4.8 mmol/L), confirming co-existing EDKA. **Results**: Following empagliflozin discontinuation, targeted treatment with concurrent insulin–dextrose infusion resulted in complete resolution of acid-base imbalance within five days. **Conclusions**: A concurrent surgical complication appeared to mask EDKA and contributed to a delay in its recognition. In patients receiving SGLT-2 inhibitors, metabolic acidosis that persists after an apparent surgical cause has been addressed should prompt measurement of serum ketones, irrespective of the blood glucose concentration. Structured perioperative protocols for SGLT-2 inhibitor management and postoperative ketone surveillance may help to prevent similar events.

## 1. Introduction

Sodium–glucose co-transporter-2 (SGLT-2) inhibitors have emerged as a cornerstone pharmacotherapy for type 2 diabetes mellitus (T2DM), with strong evidence showing their effectiveness in reducing cardiovascular mortality, heart failure, hospitalizations, and the progression of chronic kidney disease [1,2]. Empagliflozin, dapagliflozin, and canagliflozin are among the most commonly prescribed SGLT-2 inhibitors, and their use is increasingly noted in patients undergoing elective surgery.

Euglycemic diabetic ketoacidosis (EDKA) is a serious yet often overlooked complication of SGLT-2 inhibitor therapy. Unlike classic diabetic ketoacidosis (DKA), EDKA occurs with significant ketoacidosis while blood glucose levels remain near normal (typically < 250 mg/dL), making standard glycemia-based screening inadequate for early detection [3,4]. The perioperative period poses a particularly high risk for EDKA, due to factors such as surgical fasting, catecholamine-mediated stress responses, and the direct effects of SGLT-2 inhibitors on urinary ketone excretion [5,6].

Current guidelines from the American Diabetes Association (ADA) and the European Society of Anaesthesiology and Intensive Care (ESAIC) recommend discontinuing SGLT-2 inhibitors at least 3–4 days prior to elective procedures [7,8]. However, awareness and adherence to these guidelines remain inconsistent in clinical practice, partly due to the lack of structured institutional protocols [9]. A 2026 multidisciplinary consensus statement from the Society for Perioperative Assessment and Quality Improvement (SPAQI) has recently summarized the variability between existing recommendations, proposing an individualized approach to the perioperative management of these agents together with explicit strategies for the monitoring and prevention of EDKA [10].

We present a case where the diagnosis of perioperative EDKA was significantly delayed because a concurrent surgical complication—pneumoperitoneum resulting from Hemovac drain misplacement—offered a similarly compelling but ultimately insufficient explanation for the patient’s metabolic acidosis. This case highlights a critical diagnostic pitfall and emphasizes the need for systematic management protocols for SGLT-2 inhibitors in the perioperative setting.

## 2. Detailed Case Description

### 2.1. Patient Information and Preoperative Evaluation

A 71-year-old male (body mass index 24.8 kg/m^2^; height 146 cm; body weight 52.9 kg) with T2DM (HbA1c 7.2%), hypertension, and hyperlipidemia was admitted for elective direct lateral interbody fusion (DLIF) at the L4–5 level to address lumbar sacral radiculopathy. His anti-diabetic regimen included empagliflozin 10 mg/day, which was continued without interruption. Preoperative laboratory evaluations conducted 28 days prior to surgery showed that electrolytes, renal function, and blood glucose levels were within acceptable limits. With respect to glycemic control, HbA1c was 7.2% and fasting plasma glucose was 112 mg/dL. Postprandial blood glucose is not part of the routine preoperative work-up at our institution and was therefore not measured; serum and urinary ketones were likewise not assessed preoperatively. There was no institutional protocol for the discontinuation of SGLT-2 inhibitors before surgery, so the medication was not withheld. The American Society of Anesthesiologists (ASA) physical status was classified as II, and preoperative vital signs were within normal limits. The patient was fasted from midnight, giving a preoperative fasting interval of more than 8 h before induction of anesthesia.

### 2.2. Intraoperative Course

General anesthesia was induced with propofol at a dose of 1.5 mg/kg and rocuronium at 0.6 mg/kg and maintained with sevoflurane in an oxygen-air mixture with an FiO_2_ of 0.4. Intraoperative analgesia was provided with a continuous infusion of remifentanil delivered at 0.08–0.15 μg/kg/min and titrated against blood pressure and heart rate. Standard monitoring included three-lead electrocardiography, non-invasive blood pressure, pulse oximetry, end-tidal CO_2_, and bispectral index. The patient remained hemodynamically stable throughout the procedure. The operative time was approximately 210 min, with an estimated blood loss of 350 mL. Intraoperative fluid therapy consisted of 1550 mL of a balanced crystalloid solution (Plasma Solution A Inj.; HK inno.N, Cheongju, Chungcheongbuk-do, Republic of Korea); no colloid, blood product, or glucose-containing solution was given. Intraoperative urine output was 460 mL. At the conclusion of surgery, a closed-suction Hemovac drain was inserted via the left subcostal approach. The patient was extubated without complications and transferred to the post-anesthesia care unit in stable condition.

### 2.3. Postoperative Course and Diagnosis

The immediate postoperative period was unremarkable. However, on postoperative day 2, progressive altered mental status with drowsiness and low-grade fever (38.2 °C) were noted. On examination, the patient was drowsy but arousable and oriented to person and place. The abdomen was mildly distended with diffuse tenderness on palpation, but there was no guarding, rigidity, or rebound tenderness, and bowel sounds were decreased. This combination of altered mental status, fever, and abnormal abdominal findings prompted urgent laboratory testing and imaging. Urgent arterial blood gas analysis (ABGA) demonstrated severe high anion-gap metabolic acidosis: pH 7.204, PaCO_2_ 26.5 mmHg, HCO_3_^−^ 10.1 mEq/L, base excess −16.5 mEq/L, and anion gap 22 mEq/L, with a blood glucose of 178 mg/dL (Table 1). Arterial lactate was only mildly elevated at 3.0 mmol/L and therefore accounted for a small fraction of the elevated anion gap (Table 2). Inflammatory markers were significantly elevated (white blood cell count 18,200/μL, C-reactive protein 24.6 mg/dL, procalcitonin 3.8 ng/mL).

**Figure 1:**
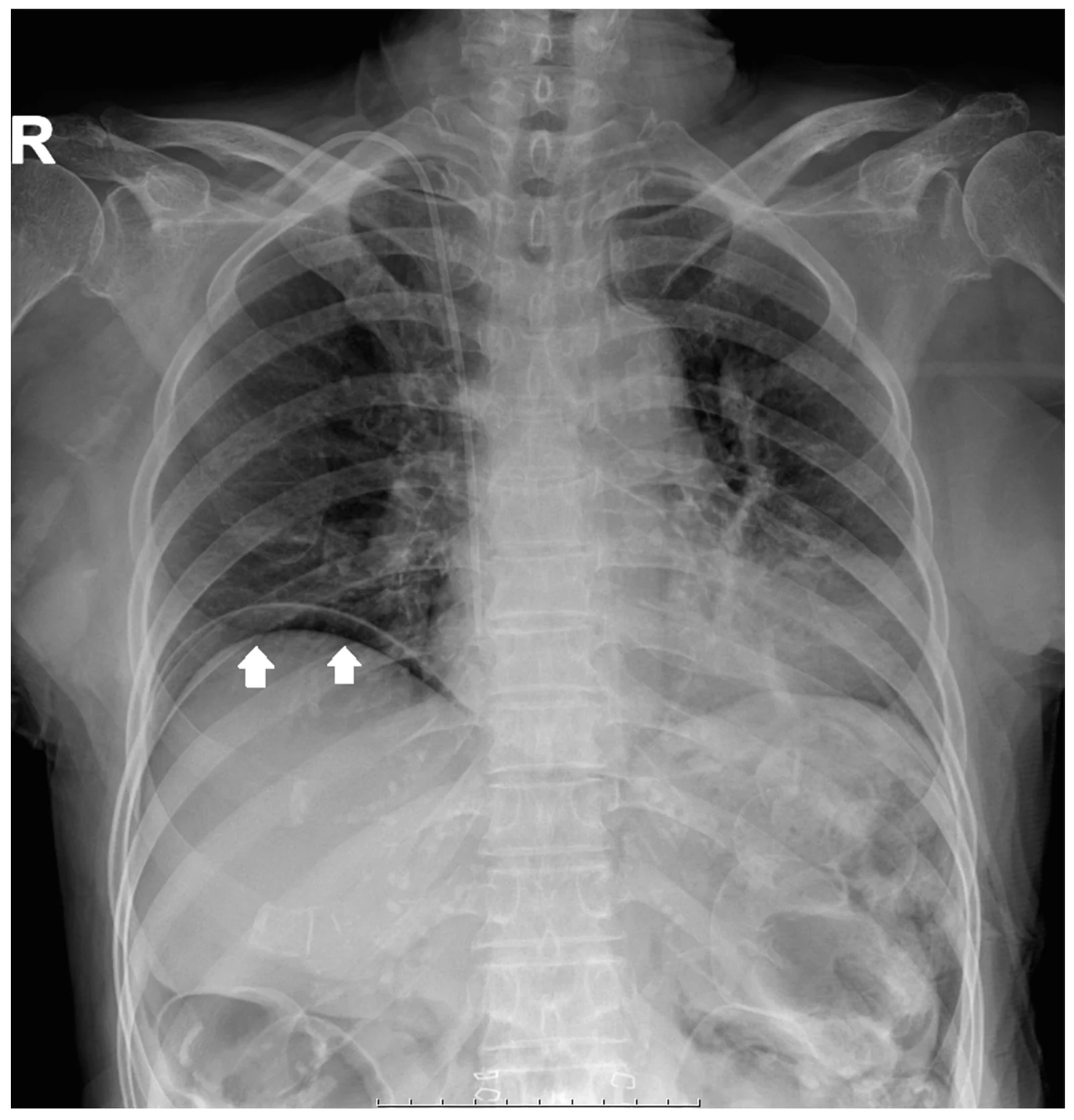
Portable anteroposterior chest radiograph obtained on postoperative day 2, demonstrating free air beneath the left hemidiaphragm (arrows), indicative of pneumoperitoneum. ‘R’ indicates the patient’s right side.

To determine the etiology of the metabolic acidosis, plain chest and abdominal radiographs were obtained, revealing free intraperitoneal air (Figure 1). Emergent contrast-enhanced computed tomography (CT) scan of the abdomen and pelvis confirmed a moderate amount of pneumoperitoneum in the bilateral subphrenic spaces and left paracolic gutter, extending posteriorly into the left posterior pararenal space. The distribution of free air followed the lateral surgical approach tract and the left flank Hemovac insertion site. While no definitive bowel perforation was identified on the CT scan, microperforation of adjacent bowel could not be excluded. Additional findings included intramuscular air in the bilateral psoas muscles, subcutaneous emphysema along the left lateral abdominal wall, mild paralytic ileus, and incidental sigmoid diverticulosis.

**Figure 2:**
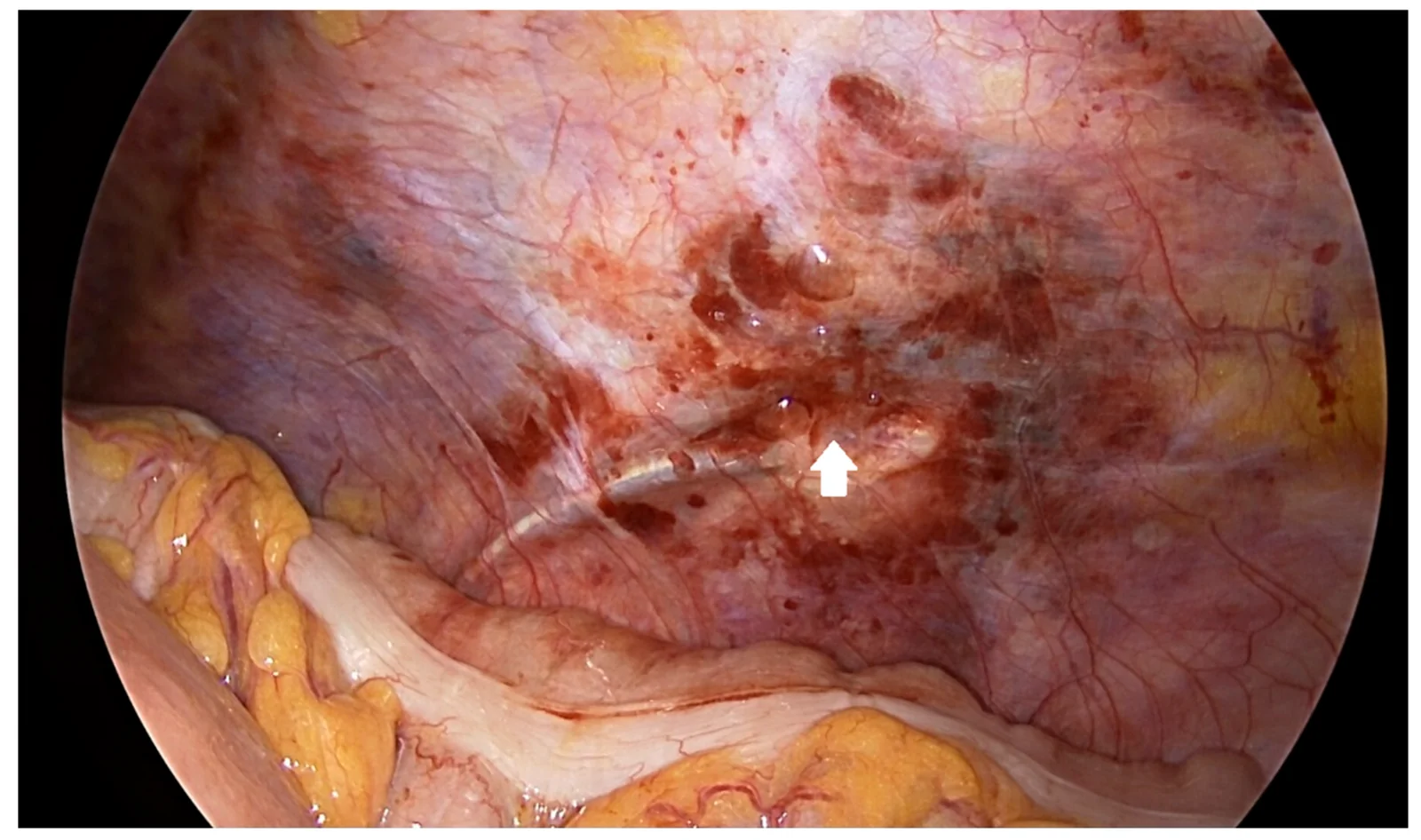
Intraoperative photograph obtained during laparoscopic exploration, demonstrating the Hemovac drain (white tubular structure, center) traversing the left subcostal peritoneum with surrounding hemorrhagic peritoneal laceration (arrow). No bowel perforation was identified. This finding confirmed the drain as the mechanical source of pneumoperitoneum.

Given the combination of high anion-gap metabolic acidosis, systemic inflammation, and pneumoperitoneum, surgical sepsis was established as the working diagnosis. Because bowel microperforation could not be excluded radiologically and the patient was clinically deteriorating, urgent surgical exploration was considered mandatory. General surgery was consulted emergently, and the patient underwent laparoscopic exploration on postoperative day 3. Intraoperatively, no bowel perforation was identified. It was confirmed that the Hemovac drain had traversed the peritoneal cavity at the left subcostal region, resulting in a partial peritoneal laceration at the insertion site (Figure 2). Drain removal and primary peritoneal repair were performed, and broad-spectrum antibiotics were initiated.

### 2.4. Diagnostic Reassessment and EDKA Diagnosis

Despite successful surgical source control and antibiotic therapy, serial ABGA demonstrated persistent high anion-gap metabolic acidosis. Post-laparoscopy ABGA (POD #4) revealed pH 7.275, PaCO_2_ 26.8 mmHg, HCO_3_^−^ 12.0 mEq/L, and base excess −13.3 mEq/L, with lactate that had fallen only marginally to 3.8 mmol/L—a clinical course inconsistent with isolated sepsis following adequate source control (Table 1 and Table 2).

At this point, the clinical team reevaluated the differential diagnosis. Given the patient’s continuous use of empagliflozin and the persistence of near-normal blood glucose levels despite severe acidosis, SGLT-2 inhibitor-associated EDKA was suspected. Serum beta-hydroxybutyrate was measured and found to be markedly elevated at 4.8 mmol/L, confirming the diagnosis of EDKA.

In retrospect, several factors account for the fact that EDKA was not suspected earlier. First, blood glucose remained near normal throughout, so the glucose-based screening that anchors most bedside assessments of diabetic emergencies never triggered concern. Second, a radiologically confirmed alternative explanation for the acidosis was available, supported by leukocytosis, a C-reactive protein of 24.6 mg/dL and a procalcitonin of 3.8 ng/mL, all of which are readily attributed to an intra-abdominal source. Third, serum ketones were not part of the standard postoperative laboratory panel at our institution and were therefore not available during the first day of the illness. Finally, the elevated anion gap of 22 mEq/L was only partly explained by the modest hyperlactatemia (3.0–3.9 mmol/L); this residual unmeasured anion, in a patient taking an SGLT-2 inhibitor, should itself have prompted earlier ketone testing. Had beta-hydroxybutyrate been measured at the time of the index ABGA on postoperative day 2, the diagnosis could have been established approximately 33 h earlier and specific treatment could have been started in parallel with, rather than after, surgical management.

### 2.5. Treatment of EDKA and Outcome

Concurrent intravenous regular insulin infusion (0.05 units/kg/h) and a 10% dextrose solution were initiated to achieve ketone clearance while preventing hypoglycemia, in accordance with EDKA management protocols [11,12]. Aggressive intravenous fluid resuscitation with 0.9% normal saline was administered, and serial ABGA and serum beta-hydroxybutyrate levels were monitored every four hours.

Serum beta-hydroxybutyrate fell from 4.8 mmol/L at diagnosis to 3.7 mmol/L on the following day, and the anion gap narrowed progressively. Biochemical criteria for resolution of ketoacidosis (pH > 7.30 and serum bicarbonate > 18 mEq/L, with normalization of the anion gap) were met by postoperative day 6 (pH 7.435, HCO_3_^−^ 31.2 mEq/L), and acid–base status was fully normalized on postoperative day 8 (pH 7.465, PaCO_2_ 38.8 mmHg, HCO_3_^−^ 27.5 mEq/L) (Table 1 and Table 2). Arterial lactate, which had been mildly elevated during the acute phase (peak 3.9 mmol/L), also returned to normal (1.2 mmol/L on postoperative day 6), supporting ketoacidosis rather than hypoperfusion as the dominant mechanism. Blood glucose levels remained within the range of 130–210 mg/dL throughout the treatment period. The patient’s clinical status gradually improved, leading to his transfer from the intensive care unit to the general ward on postoperative day 5. He was ultimately discharged on postoperative day 12 in stable condition, with diabetic management transitioned to insulin therapy following an endocrinology consultation prior to discharge.

## 3. Discussion

This case illustrates a diagnostic scenario in which EDKA may be obscured by a concurrent surgical complication. In our patient, the identification of pneumoperitoneum provided a plausible physiological explanation for the metabolic acidosis, effectively shifting attention away from considering EDKA as a potential co-existing diagnosis. We wish to emphasize that this clinical assessment was not unreasonable; given the radiologic uncertainty regarding bowel microperforation and the overall clinical picture of systemic inflammation, the decision to proceed with diagnostic laparoscopy was justifiable and could not safely have been deferred pending metabolic investigation. The learning point of this case is therefore not that the operation should have been avoided, but that ketone testing should have been performed in parallel with, rather than after, surgical management.

The pathophysiology of SGLT-2 inhibitor-induced EDKA in the perioperative context is multifactorial [3,13]. SGLT-2 inhibitors promote glucosuria independently of insulin, leading to chronic suppression of plasma insulin and an increase in glucagon levels, which in turn stimulates lipolysis and hepatic ketogenesis. Perioperative fasting, catecholamine release from surgical stress, and carbohydrate restriction further enhance ketone production [4,14]. Meanwhile, SGLT-2 inhibitors reduce renal ketone excretion, accelerating their accumulation. Importantly, the increased glucosuria helps maintain near-normal blood glucose levels, resulting in the characteristic euglycemic presentation that can mislead clinicians who rely on glycemia as a DKA screening parameter [15,16].

The half-life of SGLT-2 inhibitors ranges from approximately 11 to 13 h; however, the pharmacodynamic effects on urinary glucose excretion may persist for 2 to 4 days after discontinuation [17]. This highlights the importance of current guideline recommendations to discontinue these medications at least 3 to 4 days prior to elective surgery [7,8]. In our patient, the lack of an institutional protocol directly contributed to the continuation of the drug and the subsequent development of EDKA.

The turning point in this case was the persistence of high anion-gap metabolic acidosis, with only a marginal fall in lactate, after the mechanical source of the pneumoperitoneum had been addressed—a course that is difficult to reconcile with sepsis alone but is consistent with ongoing ketogenesis [18].

This case has several important broader implications. First, surgical complications and EDKA are not mutually exclusive diagnoses. Clinicians must remain aware that EDKA can coexist with—and be masked by—concurrent inflammatory or infectious processes. The assumption that correcting the surgical problem will also correct the metabolic abnormality may therefore be misleading in patients taking SGLT-2 inhibitors. In this population, if metabolic acidosis does not improve after the surgical cause has been addressed, serum beta-hydroxybutyrate should be measured promptly, irrespective of the blood glucose concentration [19].

Second, the treatment of SGLT-2 inhibitor-associated EDKA differs significantly from classic DKA management [11,12]. Because blood glucose levels are near normal, insulin infusion cannot be titrated to glycemia in the conventional manner. Concurrent dextrose administration is mandatory from the outset. The therapeutic endpoint is ketone clearance (beta-hydroxybutyrate < 0.6 mmol/L) and acid-base normalization, rather than glycemic control. Premature discontinuation of insulin infusion based solely on blood glucose levels risks the recurrence of ketoacidosis.

Third, this case highlights the potential value of institutional systems. The lack of a preoperative SGLT-2 inhibitor screening protocol may have contributed to drug continuation and the subsequent cascade of complications. Recognizing that a single case cannot establish the effectiveness of any particular pathway, we suggest that institutions consider perioperative SGLT-2 inhibitor management protocols comprising: (1) preoperative identification and discontinuation 3–4 days before elective surgery or according to the applicable national or society recommendation [7,8,20]; (2) enhanced postoperative monitoring, including serial ketone measurement, for patients presenting without adequate washout; (3) a diagnostic algorithm specifying that serum beta-hydroxybutyrate should be measured promptly in any SGLT-2 inhibitor user with perioperative metabolic acidosis, regardless of blood glucose level; and (4) clinical education emphasizing that EDKA and surgical complications are not mutually exclusive, and that persistent acidosis after the surgical cause has been addressed should raise suspicion of coexisting EDKA.

The anesthesia team’s role in preoperative medication reconciliation is particularly important. The preoperative anesthesia evaluation serves as an ideal and often final checkpoint for identifying SGLT-2 inhibitor use, counseling patients on discontinuation requirements, and implementing enhanced monitoring plans for high-risk patients presenting without adequate washout [21]. Systematic integration of SGLT-2 inhibitor screening into standardized preoperative checklists—including electronic medical record-based alerts—represents an actionable systems-level intervention, although its effect on the incidence of perioperative EDKA remains to be demonstrated.

**Figure 3:**
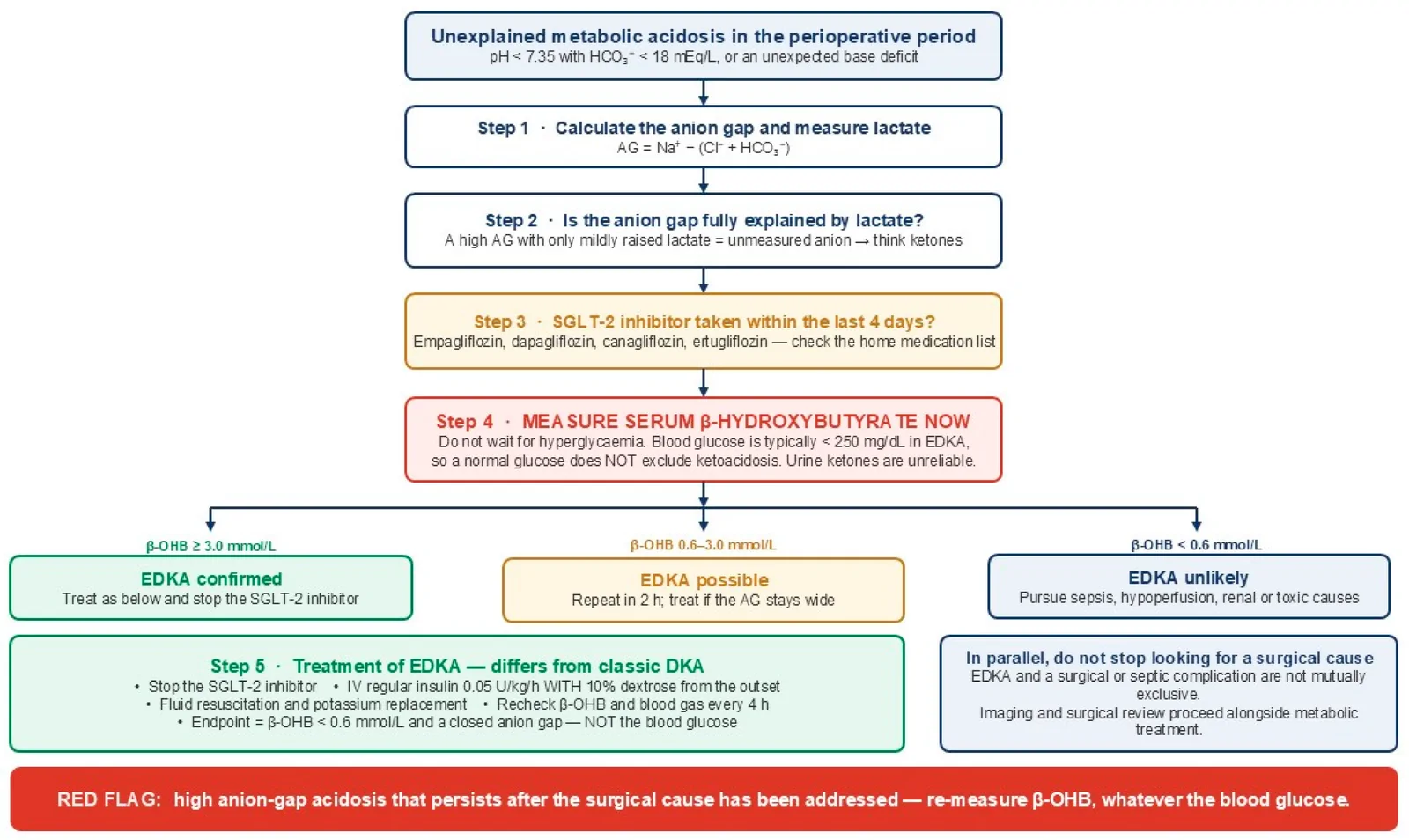
Bedside algorithm for the evaluation of perioperative high anion-gap metabolic acidosis in patients receiving SGLT-2 inhibitors. The central principle is that serum beta-hydroxybutyrate should be measured whenever unexplained high anion-gap metabolic acidosis occurs in a patient exposed to an SGLT-2 inhibitor, regardless of the blood glucose concentration, and that a surgical and a metabolic cause may coexist. AG, anion gap; β-OHB, beta-hydroxybutyrate; EDKA, euglycemic diabetic ketoacidosis; SGLT-2i, sodium–glucose co-transporter-2 inhibitor.

To translate these observations into practice, we propose a simple bedside algorithm for the assessment of perioperative metabolic acidosis in patients receiving SGLT-2 inhibitors (Figure 3). The algorithm is offered as an educational aid derived from this single case and from existing consensus recommendations [7,8,9,10]; it has not been prospectively validated and is intended to prompt, rather than replace, individualized clinical judgment.

This report has several limitations. It describes a single patient, so the observations are hypothesis-generating rather than confirmatory and cannot be generalized. Serum ketones were not measured before postoperative day 4; consequently, the time of onset of ketoacidosis cannot be established, and it is not possible to determine whether EDKA preceded the surgical complication, developed concurrently with it, or arose as a secondary consequence of the associated stress response, fasting, and inflammation. All of these sequences are biologically plausible, and the two processes most likely reinforced one another. Beta-hydroxybutyrate was measured on only two occasions during treatment, so resolution was documented principally by acid–base and anion-gap criteria rather than by serial ketone concentrations. Insulin, C-peptide, and glucagon were not measured, and the relative contributions of drug exposure, fasting, and surgical stress therefore cannot be quantified. Finally, as in any retrospective single-case description, the reconstruction of the diagnostic reasoning is subject to hindsight bias.

## 4. Conclusions

We report a case of perioperative EDKA following the uninterrupted use of SGLT-2 inhibitors in a patient undergoing spinal fusion under general anesthesia. The diagnosis was delayed due to concurrent Hemovac drain-related pneumoperitoneum. The laparoscopic exploration was clinically justified, and the principal lesson is not that surgery should have been avoided but that a metabolic cause should have been sought in parallel. In patients receiving SGLT-2 inhibitors, metabolic acidosis that persists after an apparent surgical cause has been addressed should prompt measurement of serum beta-hydroxybutyrate, irrespective of the blood glucose concentration. Although conclusions drawn from a single case must remain cautious, this experience supports the implementation of structured perioperative protocols for the identification and discontinuation of SGLT-2 inhibitors and for postoperative ketone surveillance.

## Figures and Tables

**Table 1:** Summary of Clinical Timeline and Key Laboratory Parameters.

Timepoint	Clinical Event	Blood Glucose (mg/dL)	Acid-Base Status	Intervention
Preoperative	Routine labs; SGLT-2i continued	112	Normal	None
Surgery day	DLIF performed; Hemovac inserted	145	Normal	General anesthesia
POD #1	Uneventful recovery	130–180	Normal	Routine postop care
POD #2–3	Drowsiness, fever, pneumoperitoneum	178	Severe high anion-gap metabolic acidosis	Laparoscopic exploration
POD #4–5	EDKA diagnosed; Treatment started; ICU monitoring	140–180	AG normalizing; β-OHB 4.8 → 3.7	EDKA Tx; Insulin + Dextrose
POD #5	Transfer to general ward	128	Normalized	Transition to SC insulin
POD #12	Discharge	98–99	Normal	Endocrinology f/u

POD, postoperative day; DLIF, direct lateral interbody fusion; EDKA, euglycemic diabetic ketoacidosis; AG, anion gap; β-OHB, beta-hydroxybutyrate; ICU, intensive care unit; SC, subcutaneous; SGLT-2i, sodium–glucose co-transporter-2 inhibitor; Tx, treatment; f/u, follow-up.

**Table 2:** Serial arterial blood gas and lactate values and clinical context from postoperative day 2 to postoperative day 8.

Day	Time	pH	PaCO_2_ (mmHg)	HCO_3_^−^ (mEq/L)	BE (mEq/L)	Lactate (mmol/L)	Clinical Context
POD #2	18:38	7.204	26.5	10.1	−16.5	3.0	Initial ABGA—severe metabolic acidosis
	21:49	7.268	33.8	14.9	−10.7	3.8	Post-CT; pneumoperitoneum confirmed
POD #3	02:28	7.220	34.2	13.5	−13.0	3.9	Laparoscopic exploration
POD #4	03:46	7.275	26.8	12.0	−13.3	3.8	Post-laparoscopy—acidosis unchanged → EDKA diagnosed
POD #6	04:42	7.435	47.2	31.2	+6.6	1.2	EDKA treatment day 3—improving
POD #7	03:58	7.430	47.7	31.1	+6.4	1.0	Continued improvement
POD #8	07:33	7.465	38.8	27.5	+4.0	0.8	EDKA fully resolved

BE, base excess; CT, computed tomography; EDKA, euglycemic diabetic ketoacidosis.

## Data Availability

The data presented in this study are available on request from the corresponding author. The data are not publicly available due to privacy and ethical restrictions concerning patient confidentiality.

## Abbreviations

The following abbreviations are used in this manuscript:

SGLT-2	Sodium–glucose co-transporter-2
EDKA	Euglycemic diabetic ketoacidosis
T2DM	Type 2 diabetes mellitus
ABGA	Arterial blood gas analysis
AG	Anion gap
β-OHB	Beta-hydroxybutyrate
DLIF	Direct lateral interbody fusion
POD	Postoperative day
